# Supplementary material for: DNA Methylation of Imprinted Genes KCNQ1, KCNQ1OT1, and PHLDA2 in Peripheral Blood Is Associated with the Risk of Breast Cancer
Source: Cancers (Basel). 2022 May 27;14(11):2652. doi: 10.3390/cancers14112652 (PMC9179312; doi:10.3390/cancers14112652)
Supplement: Supplementary file 1 [file cancers-14-02652-s001.zip › cancers-1707104-supplementary.pdf]

Supplementary Materials and Methods

The DNA methylation levels of imprinted genes in the peripheral blood leukocyte samples were detected by MethylTarget™ (Genesky Biotechnologies Inc., Shanghai, China), a next-generation sequencing-based multiple Targeted CpG methylation analysis method. A multiplex polymerase chain reaction (PCR) was performed with optimized combination of primers. A 20 μl PCR reaction mixture was prepared for each reaction, which included 10 × reaction buffer (Takara Bio, Otsu, Japan), 25mM of Mg^{2+}, 2.5mM of dNTP, 1μM of each primer, 5U/μL HotStarTaq polymerase (Takara) and 1 μL of template DNA. PCR amplicons were diluted and amplified using indexed primers. The cycling program was 95℃ for 2 minutes; 11 cycles of 95℃ for 20 seconds, 62℃ for 40 seconds with a decreasing temperature step of 0.5℃ per cycle, 72℃ for 1 minute; then followed by 24 cycles of 95℃ for 20 seconds, 62℃ for 30 seconds, 72℃ for 1 minute; and, finally, 72℃ for 1 minute.

Libraries from different samples were quantified, pooled together, and used for sequencing on the MiSeq platform (Illumina, Inc., San Diego, CA, USA). Sequencing was performed with a 2 × 300 bp paired-end mode. Quality control of sequencing reads was performed by FastQC (http://www.bioinformatics.babraham.ac.uk/projects/fastqc/). Assembling the filtered R1 and R2 reads were conducted with FLASH (Fast length adjustment of short reads to improve genome assemblies) (http://www.cbcb.umd.edu/software/flash). FastX was used to process fastq files to obtain the fa format sequence (http://hannonlab.cshl.edu/fastx_toolkit/index.html). All reads in the fa and reference sequences of target region were compared by BLAT (http://genome.ucsc.edu/FAQ/FAQblat.html). Reads that could cover 90% of its target sequences or with 90% bases that could completely cover target sequences, which were classified as effective reads.

Table S1. Main characteristics of samples from TCGA, GEO and validation datasets.

Datasets	Sample type	Assay	Groups	No. of samples	Mean age
GSE74738	Placental chorionic villi	Illumina 450K Assay	Diandric triploid	5	11.8 (weeks)
			Digynic triploid	5	9.4 (weeks)
TCGA-BC	Tissue	Illumina 450K Assay	BC tissue	782	58.0 (years)
			Normal adjacent breast	96	57.6 (years)
GSE51032	Peripheral blood leukocytes	Illumina 450K Assay	BC patients	233	52.4 (years)
			Control	340	52.6 (years)
Inhouse study	Peripheral blood leukocytes	Targeted methylation sequencing	BC patients	514	52.0 (years)
			Control	534	52.1 (years)

TCGA, The Cancer Genome Atlas; GEO, Gene Expression Omnibus.

Table S2. Sequences of primers and products of targeted sequencing array.

Gene [a]	mRNA	Primer sequence	Primer location	Product information			
				Genomic location	Product size (bp)	Strand	CpG
CDKN1C	NM_000076	F-GTATTTGGTTTGTGGAAYGTTTAGTTYGTTTG	Chr11: 2906991-2907022	Chr11: 2906991-2907276	286	+	43
		R-CCCCCTTCCCCTACTACAAC	Chr11: 2907257-2907276				
KCNQ1OT1	NR_002728	F-GGGYGGTAGTAGGGGAAGGTTTG	Chr11: 2721298-2721320	Chr11: 2721298-2721469	172	+	11
		R-CCACCTCACACCCAACCA	Chr11: 2721452-2721469				
KCNQ1OT1	NR_002728	F-GATATGTTAGGTTATTTATTTGGTAAAGGGTAG	Chr11: 2721919-2721951	Chr11: 2721919-2722151	233	+	22
		R-AAAAAACTTTTATAACCCAAACTTTTATCC	Chr11: 2722122-2722151				
KCNQ1	NM_181798	F-TTTGGGTGGTATTGTTGTAGGATTT	Chr11: 2482191-2482215	Chr11: 2482191-2482430	240	+	7
		R-CCTAATCACACCTAACCCATCACC	Chr11: 2482407-2482430				
PHLDA2	NM_003311	F-GTAGTTYGTGGGTGGTAAGATAGGAGTT	Chr11: 2951272-2951245	Chr11: 2951272-2951065	208	-	13
		R-CCAAACCTTCACCCTCCAAA	Chr11: 2951084-2951065				

[a] All genome coordinates are based on the human genome version GRCh37/hg19.

Table S3. Differential methylation of 16 CpG sites of imprinted genes associated with the risk of breast cancer in TCGA and GEO datasets.

CpG	Gene symbol	Chromosome	Genomic coordinate	TCGA				GSE51032			
				BC tissue	Normal tissue	Δβ	*p*	BC PBLs	Control PBLs	Δβ	*p*
cg18772071	*NAP1L5*	4	89618324	0.490	0.441	0.049	<0.001	0.557	0.541	0.016	<0.001
cg21740964	*FAM50B*	6	3849331	0.540	0.574	-0.034	0.011	0.518	0.529	-0.011	0.012
cg27589003	*MEST*	7	130132286	0.467	0.427	0.040	<0.001	0.485	0.469	0.016	<0.001
cg27120649	*SGCE*	7	94286261	0.335	0.319	0.016	0.044	0.459	0.449	0.010	0.033
cg01169624	*SGCE*	7	94286936	0.593	0.640	-0.047	<0.001	0.672	0.685	-0.013	0.008
cg04402633	*CDKN1C*	11	2907113	0.067	0.055	0.012	<0.001	0.142	0.130	0.012	<0.001
cg11744767	*CDKN1C*	11	2907672	0.404	0.232	0.172	<0.001	0.315	0.304	0.011	0.019
cg04975775	*H19*	11	2019587	0.428	0.546	-0.112	<0.001	0.631	0.641	-0.010	0.018
cg15886040	*H19*	11	2019606	0.538	0.594	-0.056	<0.001	0.659	0.671	-0.012	<0.001
cg26709929	*KCNQ1*	11	2482240	0.857	0.910	-0.053	<0.001	0.862	0.872	-0.010	<0.001
cg08446215	*KCNQ1OT1*	11	2721366	0.540	0.551	-0.011	0.031	0.672	0.682	-0.010	<0.001
cg25306939	*KCNQ1OT1*	11	2722086	0.510	0.537	-0.027	0.037	0.588	0.598	-0.010	0.014
cg16057921	*PHLDA2*	11	2951168	0.058	0.046	0.012	<0.001	0.150	0.134	0.016	<0.001
cg14245102	*MEG3*	14	101292967	0.678	0.446	0.232	<0.001	0.559	0.539	0.020	<0.001
cg09596866	*ZNF597*	16	3493336	0.322	0.279	0.043	<0.001	0.510	0.490	0.019	0.007
cg12054318	*GNASAS*	20	57414529	0.485	0.547	-0.062	<0.001	0.606	0.629	-0.023	<0.001

BC, breast cancer; PBLs, peripheral blood leukocytes, TCGA, The Cancer Genome Atlas; GEO, Gene Expression Omnibus. Δβ represented the mean difference in methylation between BC tissues and normal tissues from TCGA, and between BC PBLs and control PBLs from GEO dataset (GSE51032).

Table S4. Demographic characteristics of breast cancer patients and controls for *KCNQ1* region sequencing.

Characteristics	Case (%)	Control (%)	*p*
Number of participants	494 (48.5)	525 (51.5)	
Age (year) [a, b]	52.0 (9.36)	52.1 (10.80)	0.939
<50	208 (42.2)	235 (44.8)	0.002
50-	184 (37.2)	142 (27.0)	
60-	84 (17.0)	120 (22.9)	
≥70	18 (3.6)	28 (5.3)	
BMI (kg/m^2) [a, b]	23.9 (3.64)	23.7 (4.11)	0.373
<18.5	15 (3.0)	27 (5.1)	0.332
18.5-	257 (52.0)	266 (50.7)	
24-	133 (26.9)	133 (26.9)	
≥28	89 (18.1)	89 (18.0)	
Ethnicity [b]			0.279
Han	482 (97.6)	505 (96.2)	
Other	12 (2.4)	20 (3.8)	
Location [b]			0.484
City	400 (81.0)	435 (82.9)	
Rural area	94 (19.0)	90 (17.1)	
Family history of other cancer [b]			0.001
No	377 (76.3)	445 (84.8)	
Yes	117 (23.7)	80 (15.2)	
Family history of breast cancer [b]			<0.001
No	456 (92.3)	517 (98.5)	
Yes	38 (7.7)	8 (1.5)	

[a] mean (standard deviation), [b] n (%).

Table S5. Demographic characteristics of breast cancer patients and controls for *KCNQ1OT1* region 1 sequencing.

**Characteristics**	**Case (%)**	**Control (%)**	***p***
Number of participants	510 (48.9)	533 (51.1)	
Age (year) [a, b]	52.0 (9.35)	52.1 (10.75)	0.925
<50	214 (42.0)	237 (44.5)	0.004
50-	190 (37.3)	147 (27.6)	
60-	88 (17.3)	121 (22.7)	
≥70	18 (3.4)	28 (5.3)	
BMI (kg/m^2) [a, b]	23.9 (3.66)	23.7 (4.10)	0.363
<18.5	15 (2.9)	27 (5.1)	0.327
18.5-	269 (52.7)	271 (50.8)	
24-	134 (26.3)	133 (25.0)	
≥28	92 (18.0)	102 (19.1)	
Ethnicity [b]			0.198
Han	498 (97.6)	512 (96.1)	
Other	12 (2.4)	21 (3.9)	
Location [b]			0.512
City	414 (81.2)	442 (82.9)	
Rural area	96 (18.8)	91 (17.1)	
Family history of other cancer [b]			<0.001
No	388 (76.1)	453 (85.0)	
Yes	122 (23.9)	80 (15.0)	
Family history of breast cancer [b]			<0.001
No	470 (92.2)	525 (98.5)	
Yes	40 (7.8)	8 (1.5)	

[a] mean (standard deviation), [b] n (%).

Table S6. Demographic characteristics of breast cancer patients and controls for *KCNQ1OT1* region 2 sequencing.

**Characteristics**	**Case (%)**	**Control (%)**	***p***
Number of participants	354 (53.4)	309 (46.6)	
Age (year) [a, b]	52.2 (9.44)	52.3 (10.94)	0.869
<50	147 (41.5)	139 (45.0)	0.092
50-	128 (36.2)	86 (27.8)	
60-	64 (18.1)	63 (20.4)	
≥70	15 (4.2)	21 (6.8)	
BMI (kg/m^2) [a, b]	23.8 (3.54)	23.7 (4.38)	0.814
<18.5	12 (3.4)	15 (4.9)	0.389
18.5-	182 (51.4)	163 (52.8)	
24-	98 (27.7)	70 (22.6)	
≥28	62 (17.5)	61 (19.7)	
Ethnicity [b]			0.790
Han	347 (98.0)	301 (97.4)	
Other	7 (2.0)	8 (2.6)	
Location [b]			0.761
City	289 (81.6)	256 (82.8)	
Rural area	65 (18.4)	53 (17.2)	
Family history of other cancer [b]			0.003
No	274 (77.4)	268 (86.7)	
Yes	80 (22.6)	41 (13.3)	
Family history of breast cancer [b]			<0.001
No	330 (93.2)	307 (99.4)	
Yes	24 (6.8)	2 (0.6)	

[a] mean (standard deviation), [b] n (%).

Table S7. Demographic characteristics of breast cancer patients and controls for *PHLDA2* region sequencing.

**Characteristics**	**Case (%)**	**Control (%)**	***p***
Number of participants	501 (48.6)	529 (51.4)	
Age (year) [a, b]	52.0 (9.35)	52.1 (10.79)	0.920
<50	211 (42.1)	236 (44.6)	0.004
50-	186 (37.1)	145 (27.4)	
60-	86 (17.2)	120 (22.7)	
≥70	18 (3.6)	28 (5.3)	
BMI (kg/m^2) [a, b]	23.9 (3.67)	23.7 (4.09)	0.367
<18.5	15 (3.0)	26 (4.9)	0.410
18.5-	261 (52.1)	270 (51.0)	
24-	134 (26.7)	132 (25.0)	
≥28	91 (18.2)	101 (19.1)	
Ethnicity [b]			0.209
Han	489 (97.6)	508 (96.0)	
Other	12 (2.4)	21 (4.0)	
Location [b]			0.570
City	408 (81.4)	439 (83.0)	
Rural area	93 (18.6)	90 (17.0)	
Family history of other cancer [b]			0.001
No	383 (76.4)	449 (84.9)	
Yes	118 (23.6)	80 (15.1)	
Family history of breast cancer [b]			<0.001
No	462 (92.2)	521 (98.5)	
Yes	39 (7.8)	8 (1.5)	

[a] mean (standard deviation), [b] n (%).

Table S8. The association between the methylation of CpG sites on *KCNQ1* and the risk of breast cancer.

			Univariate analysis		Multivariate analysis	
**CpG_Position**	**Δβ**	***p* (t-test)**	**OR (95% CI)**	***p***	**OR (95% CI)** [a]	***p***
*KCNQ1*_2482223	-0.001	0.267	0.921 (0.773-1.052)	0.289	0.913 (0.764-1.045)	0.249
*KCNQ1*_2482233	-0.001	0.029	0.871 (0.768-0.985)	0.029	0.870 (0.765-0.989)	0.033
*KCNQ1*_2482240 [b]	-0.002	0.049	0.859 (0.731-0.990)	0.048	0.852 (0.723-0.985)	0.047
*KCNQ1*_2482284	-0.003	0.129	0.909 (0.803-1.028)	0.129	0.907 (0.797-1.030)	0.134
*KCNQ1*_2482303	-0.002	0.357	0.944 (0.834-1.067)	0.356	0.938 (0.825-1.066)	0.329
*KCNQ1*_2482394	-0.001	0.499	0.958 (0.842-1.085)	0.504	0.950 (0.831-1.079)	0.431
*KCNQ1*_2482401	-0.001	0.532	0.961 (0.849-1.087)	0.530	0.948 (0.833-1.077)	0.411

OR, odds ratio; 95% CI, 95% confidence intervals. Δβ represented the mean difference in methylation between breast cancer patients and controls from the validation dataset, and the ORs were calculated based on the methylation levels of CpG sites increased per standard deviation.

[a] ORs adjusted for age, BMI, race, location, family history of other cancer and family history of breast cancer.

[b] CpG site corresponding to cg26709929.

Table S9. The association between the methylation of CpG sites on *KCNQ1OT1* region 1 and the risk of breast cancer.

			Univariate analysis		Multivariate analysis	
**CpG_Position**	**Δβ**	***p* (t-test)**	**OR (95% CI)**	***p***	**OR (95% CI) [a]**	***p***
*KCNQ1OT1*_2721321	-0.001	0.835	0.987 (0.874-1.115)	0.835	0.981 (0.867-1.111)	0.766
*KCNQ1OT1*_2721324	-0.001	0.631	0.971 (0.859-1.096)	0.632	0.969 (0.855-1.096)	0.614
*KCNQ1OT1*_2721336	-0.001	0.725	0.978 (0.866-1.105)	0.726	0.972 (0.858-1.100)	0.654
*KCNQ1OT1*_2721351	-0.001	0.787	0.983 (0.870-1.111)	0.787	0.978 (0.864-1.107)	0.728
*KCNQ1OT1*_2721366 [b]	-0.001	0.824	0.986 (0.873-1.114)	0.824	0.980 (0.865-1.109)	0.747
*KCNQ1OT1*_2721383	-0.001	0.604	0.968 (0.857-1.093)	0.604	0.964 (0.850-1.090)	0.558
*KCNQ1OT1*_2721409	-0.001	0.581	0.966 (0.855-1.091)	0.581	0.962 (0.849-1.089)	0.539
*KCNQ1OT1*_2721428	-0.002	0.399	0.949 (0.839-1.072)	0.399	0.946 (0.835-1.071)	0.384
*KCNQ1OT1*_2721437	-0.002	0.357	0.944 (0.835-1.067)	0.358	0.943 (0.832-1.067)	0.353
*KCNQ1OT1*_2721440	-0.002	0.374	0.946 (0.837-1.069)	0.375	0.945 (0.833-1.069)	0.369

OR, odds ratio; 95% CI, 95% confidence intervals. Δβ represented the mean difference in methylation between breast cancer patients and controls from the validation dataset, and the ORs were calculated based on the methylation levels of CpG sites increased per standard deviation.

[a] ORs adjusted for age, BMI, race, location, family history of other cancer and family history of breast cancer.

[b] CpG site corresponding to cg08446215.

Table S10. The association between the methylation of CpG sites on *PHLDA2* region and the risk of breast cancer.

			Univariate analysis		Multivariate analysis	
**CpG_Position**	**Δβ**	***p* (t-test)**	**OR (95% CI)**	***p***	**OR (95% CI)** [a]	***p***
*PHLDA2*_2951086	0.001	0.548	1.038 (0.919-1.174)	0.548	1.050 (0.929-1.193)	0.421
*PHLDA2*_2951106	0.001	0.463	1.047 (0.927-1.184)	0.461	1.040 (0.915-1.177)	0.564
*PHLDA2*_2951112	0.001	0.648	1.029 (0.910-1.164)	0.647	1.040 (0.916-1.176)	0.563
*PHLDA2*_2951120	0.001	0.355	1.059 (0.937-1.198)	0.355	1.060 (0.931-1.197)	0.397
*PHLDA2*_2951137	0.001	0.040	1.139 (1.007-1.293)	0.041	1.150 (1.010-1.300)	0.036
*PHLDA2*_2951158	0.001	0.419	1.052 (0.931-1.190)	0.419	1.060 (0.934-1.201)	0.374
*PHLDA2*_2951168 [b]	0.002	0.034	1.144 (1.011-1.298)	0.035	1.140 (1.010-1.300)	0.041
*PHLDA2*_2951174	0.001	0.646	1.029 (0.911-1.163)	0.645	1.020 (0.898-1.155)	0.779
*PHLDA2*_2951183	0.001	0.889	1.009 (0.893-1.140)	0.888	1.000 (0.884-1.135)	0.980
*PHLDA2*_2951202	-0.001	0.994	1.000 (0.884-1.130)	0.994	1.020 (0.901-1.158)	0.744
*PHLDA2*_2951219	-0.004	0.235	0.928 (0.820-1.049)	0.234	0.935 (0.825-1.059)	0.293
*PHLDA2*_2951239	-0.001	0.659	0.973 (0.860-1.099)	0.659	0.988 (0.872-1.119)	0.849
*PHLDA2*_2951244	-0.001	0.787	0.983 (0.870-1.111)	0.787	0.996 (0.879-1.129)	0.947

OR, odds ratio; 95% CI, 95% confidence intervals. Δβ represented the mean difference in methylation between breast cancer patients and controls from the validation dataset, and the ORs were calculated based on the methylation levels of CpG sites increased per standard deviation.

[a] ORs adjusted for age, BMI, race, location, family history of other cancer and family history of breast cancer.

[b] CpG site corresponding to cg16057921.

Table S11. Detail information of cut-off value of each detected CpG sites.

CpG_Position	Cut-off value	AUC	Youden
*KCNQ1*_2482233	0.961	0.473	0.018
*KCNQ1*_2482240	0.940	0.527	0.065
*KCNQ1OT1*_2721952	0.463	0.545	0.124
*KCNQ1OT1*_2721955	0.475	0.548	0.137
*KCNQ1OT1*_2721961	0.476	0.548	0.132
*KCNQ1OT1*_2721966	0.473	0.546	0.129
*KCNQ1OT1*_2721968	0.480	0.546	0.127
*KCNQ1OT1*_2721978	0.475	0.550	0.145
*KCNQ1OT1*_2721982	0.478	0.550	0.139
*KCNQ1OT1*_2721986	0.470	0.547	0.136
*KCNQ1OT1*_2721992	0.479	0.550	0.141
*KCNQ1OT1*_2722019	0.471	0.545	0.127
*KCNQ1OT1*_2722023	0.472	0.549	0.135
*KCNQ1OT1*_2722036	0.480	0.548	0.131
*KCNQ1OT1*_2722038	0.489	0.549	0.150
*KCNQ1OT1*_2722045	0.471	0.549	0.141
*KCNQ1OT1*_2722062	0.473	0.544	0.135
*KCNQ1OT1*_2722071	0.479	0.546	0.128
*KCNQ1OT1*_2722073	0.480	0.548	0.147
*KCNQ1OT1*_2722076	0.482	0.549	0.134
*KCNQ1OT1*_2722082	0.488	0.549	0.158
*KCNQ1OT1*_2722084	0.484	0.544	0.139
*KCNQ1OT1*_2722086	0.486	0.546	0.135
*KCNQ1OT1*_2722119	0.458	0.567	0.151
*PHLDA2*_2951168	0.071	0.540	0.094
*PHLDA2*_2951137	0.028	0.530	0.097
KCNQ1OT1 region	0.474	0.551	0.150

AUC, area under curve. The optimal cut-off values of methylation level were calculated using the receiver operating characteristic curve.

Table S12. The association between methylation of CpG sites on *KCNQ1* region, *KCNQ1OT1* region 2, and *PHLDA2* region and the risk of breast cancer.

				Univariate analysis		Multivariate analysis	
**CpG_Position**		**Case (%)**	**Control (%)**	**OR (95% CI)**	***p***	**OR (95% CI)** [a]	***p***
*KCNQ1*_2482233	<0.961	257 (52.0)	278 (53.0)	1.000	0.767	1.000	0.675
	≥0.961	237 (48.0)	247 (47.0)	1.040 (0.812-1.328)		1.060 (0.820-1.360)	
*KCNQ1*_2482240	<0.940	221 (44.7)	202 (38.5)	1.000	0.043	1.000	0.090
	≥0.940	273 (55.3)	323 (61.5)	0.773 (0.602-0.991)		0.802 (0.621-1.035)	
*KCNQ1OT1*_2721952	<0.463	242 (68.4)	173 (56.0)	1.000	0.001	1.000	0.002
	≥0.463	112 (31.6)	136 (44.0)	0.589 (0.428-0-808)		0.589 (0.424-0-816)	
*KCNQ1OT1*_2721955	<0.475	256 (72.3)	182 (58.9)	1.000	<0.001	1.000	0.001
	≥0.475	98 (27.7)	127 (41.1)	0.549 (0.396-0.758)		0.555 (0.395-0.775)	
*KCNQ1OT1*_2721961	<0.476	254 (71.8)	182 (58.9)	1.000	0.001	1.000	0.001
	≥0.476	100 (28.2)	127 (41.1)	0.564 (0.408-0.779)		0.562 (0.402-0.785)	
*KCNQ1OT1*_2721966	<0.473	245 (69.2)	174 (56.3)	1.000	0.001	1.000	0.001
	≥0.473	109 (30.8)	135 (43.7)	0.573 (0.416-0.788)		0.569 (0.408-0.790)	
*KCNQ1OT1*_2721968	<0.480	257 (72.6)	186 (60.2)	1.000	0.001	1.000	0.001
	≥0.480	97 (27.4)	123 (39.8)	0.571 (0.411-0.790)		0.568 (0.405-0.795)	
*KCNQ1OT1*_2721978	<0.475	254 (71.8)	177 (57.3)	1.000	<0.001	1.000	<0.001
	≥0.475	100 (28.2)	132 (42.7)	0.528 (0.382-0.728)		0.523 (0.373-0.729)	
*KCNQ1OT1*_2721982	<0.478	261 (73.7)	186 (60.2)	1.000	<0.001	1.000	0.001
	≥0.478	93 (26.3)	123 (39.8)	0.539 (0.387-0.747)		0.553 (0.393-0.775)	
*KCNQ1OT1*_2721986	<0.470	251 (70.9)	178 (57.6)	1.000	<0.001	1.000	<0.001
	≥0.470	103 (29.1)	131 (42.4)	0.558 (0.404-0.768)		0.552 (0.395-0.769)	
*KCNQ1OT1*_2721992	<0.479	265 (74.9)	188 (60.8)	1.000	<0.001	1.000	<0.001
	≥0.479	89 (25.1)	121 (39.2)	0.522 (0.374-0.726)		0.520 (0.368-0.732)	

*KCNQ1OT1*_2722019	<0.471	239 (67.5)	171 (55.3)	1.000	0.001	1.000	0.002
	≥0.471	115 (32.5)	138 (44.7)	0.596 (0.434-0.817)		0.592 (0.426-0.821)	
*KCNQ1OT1*_2722023	<0.472	239 (67.5)	168 (54.4)	1.000	0.001	1.000	0.001
	≥0.472	115 (32.5)	141 (45.6)	0.573 (0.418-0.785)		0.562 (0.404-0.779)	
*KCNQ1OT1*_2722036	<0.480	247 (70.0)	176 (57.0)	1.000	0.001	1.000	0.001
	≥0.480	107 (30.0)	133 (43.0)	0.573 (0.418-0.785)		0.575 (0.413-0.799)	
*KCNQ1OT1*_2722038	<0.489	270 (76.3)	191 (61.8)	1.000	<0.001	1.000	<0.001
	≥0.489	84 (23.7)	118 (38.2)	0.504 (0.359-0.703)		0.507 (0.357-0.716)	
*KCNQ1OT1*_2722045	<0.471	239 (67.5)	167 (54.0)	1.000	<0.001	1.000	<0.001
	≥0.471	115 (32.5)	142 (46.0)	0.566 (0.412-0.775)		0.556 (0.401-0.770)	
*KCNQ1OT1*_2722062	<0.473	244 (68.9)	172 (55.7)	1.000	<0.001	1.000	<0.001
	≥0.473	110 (31.1)	137 (44.3)	0.566 (0.411-0.777)		0.553 (0.397-0.768)	
*KCNQ1OT1*_2722071	<0.479	247 (69.8)	177 (57.3)	1.000	0.001	1.000	0.001
	≥0.479	107 (30.2)	132 (42.7)	0.581 (0.421-0.799)		0.575 (0.413-0.800)	
*KCNQ1OT1*_2722073	<0.480	248 (70.1)	171 (55.3)	1.000	<0.001	1.000	<0.001
	≥0.480	106 (29.9)	138 (44.7)	0.530 (0.384-0.728)		0.518 (0.371-0.719)	
*KCNQ1OT1*_2722076	<0.482	250 (70.6)	178 (57.6)	1.000	0.001	1.000	0.001
	≥0.482	104 (29.4)	131 (42.4)	0.565 (0.409-0.779)		0.567 (0.406-0.789)	
*KCNQ1OT1*_2722082	<0.488	261 (73.7)	179 (57.9)	1.000	<0.001	1.000	<0.001
	≥0.488	93 (26.3)	130 (42.1)	0.491 (0.353-0.680)		0.489 (0.348-0.685)	
*KCNQ1OT1*_2722084	<0.484	253 (71.5)	178 (57.6)	1.000	<0.001	1.000	<0.001
	≥0.484	101 (28.5)	131 (42.4)	0.542 (0.392-0.748)		0.531 (0.379-0.740)	
*KCNQ1OT1*_2722086	<0.486	254 (71.8)	182 (58.9)	1.000	0.001	1.000	<0.001
	≥0.486	100 (28.2)	127 (41.1)	0.564 (0.408-0.779)		0.549 (0.392-0.767)	
*KCNQ1OT1*_2722119	<0.458	254 (71.8)	175 (56.6)	1.000	<0.001	1.000	<0.001
	≥0.458	100 (28.2)	134 (43.4)	0.514 (0.372-0.709)		0.517 (0.369-0.720)	

*PHLDA2*_2951168	<0.071	305 (60.9)	366 (69.2)	1.000	0.005	1.000	0.009
	≥0.071	196 (39.1)	163 (30.8)	1.440 (1.120-1.870)		1.420 (1.090-1.840)	
*PHLDA2*_2951137	<0.028	323 (64.5)	389 (73.5)	1.000	0.002	1.000	0.002
	≥0.028	178 (35.5)	140 (26.5)	1.531 (1.174-1.998)		1.540 (1.180-2.020)	

OR, odds ratio; 95% CI, 95% confidence intervals.

[a] ORs were calculated based on the methylation levels of CpG sites increased per standard deviation, and were adjusted for age, BMI, race, location, family history of other cancer and family history of breast cancer.

Table S13. The association between methylation of cg26709929 on *KCNQ1* region and the risk of breast cancer in different subgroups.

Subgroup	*KCNQ1*_2482240	Case (%)	Control (%)	Univariate analysis		Multivariate analysis	
				OR (95% CI)	*p*	OR (95% CI) [a]	*p*
Age (year)							
<50	<0.940	84 (40.4)	88 (37.4)	1.000	0.527	1.000	0.849
	≥0.940	124 (59.6)	147 (62.6)	0.884 (0.602-1.296)		0.962 (0.645-1.436)	
≥50	<0.940	137 (47.9)	114 (39.3)	1.000	0.038	1.000	0.054
	≥0.940	149 (52.1)	176 (60.7)	0.704 (0.506-0.980)		0.720 (0.515-1.005)	
Molecular subtype							
Luminal A	<0.940	79 (46.2)	202 (38.5)	1.000	0.074	1.000	0.119
	≥0.940	92 (53.8)	323 (61.5)	0.728 (0.514-1.033)		0.755 (0.530-1.080)	
Luminal B	<0.940	76 (41.8)	202 (38.5)	1.000	0.435	1.000	0.621
	≥0.940	106 (58.2)	323 (61.5)	0.872 (0.620-1.230)		0.914 (0.641-1.308)	
HER-2	<0.940	33 (53.2)	202 (38.5)	1.000	0.027	1.000	0.036
	≥0.940	29 (46.8)	323 (61.5)	0.550 (0.322-0.932)		0.559 (0.323-0.962)	
Basal-like	<0.940	25 (45.5)	202 (38.5)	1.000	0.314	1.000	0.470
	≥0.940	30 (54.5)	323 (61.5)	0.750 (0.430-1.320)		0.810 (0.458-1.448)	

OR, odds ratio; 95% CI, 95% confidence intervals.

[a] ORs were calculated based on the methylation levels of CpG sites increased per standard deviation, and were adjusted for age, BMI, race, location, family history of other cancer and family history of breast cancer.

Table S14. The association between methylation of the CpG located on genomic coordinate 2482233 in *KCNQ1* and the risk of breast cancer in different subgroups.

				Univariate analysis		Multivariate analysis	
**Subgroup**	***KCNQ1*_2482233**	**Case (%)**	**Control (%)**	**OR (95% CI)**	***p***	**OR (95% CI) [a]**	***p***
Age (year)							
<50	<0.961	99 (47.6)	112 (47.7)	1.000	0.989	1.000	0.676
	≥0.961	109 (52.4)	123 (52.3)	1.000 (0.690-1.460)		1.090 (0.736-1.607)	
≥50	<0.961	158 (55.2)	166 (57.2)	1.000	0.629	1.000	0.644
	≥0.961	128 (44.8)	124 (42.8)	1.080 (0.780-1.510)		1.080 (0.776-1.508)	
Molecular subtype							
Luminal A	<0.961	97 (56.7)	278 (53.0)	1.000	0.368	1.000	0.589
	≥0.961	74 (43.3)	247 (47.0)	0.859 (0.605-1.214)		0.907 (0.635-1.293)	
Luminal B	<0.961	80 (44.0)	278 (53.0)	1.000	0.037	1.000	0.040
	≥0.961	102 (56.0)	247 (47.0)	1.440 (1.020-2.020)		1.450 (1.020-2.060)	
HER-2	<0.961	36 (58.1)	278 (53.0)	1.000	0.446	1.000	0.388
	≥0.961	26 (41.9)	247 (47.0)	0.813 (0.473-1.380)		0.786 (0.451-1.352)	
Basal-like	<0.961	32 (58.2)	278 (53.0)	1.000	0.460	1.000	0.593
	≥0.961	23 (41.8)	247 (47.0)	0.809 (0.456-1.414)		0.855 (0.478-1.512)	

OR, odds ratio; 95% CI, 95% confidence intervals.

[a] ORs were calculated based on the methylation levels of CpG sites increased per standard deviation, and were adjusted for age, BMI, race, location, family history of other cancer and family history of breast cancer.

Table S15. The association between methylation of cg16057921 on *PHLDA2* region and the risk of breast cancer in different subgroups.

Subgroup	*PHLDA2*_2951168	Case (%)	Control (%)	Univariate analysis		Multivariate analysis	
				OR (95% CI)	*p*	OR (95% CI) [a]	*p*
Age (year)							
<50	<0.071	131 (62.1)	159 (67.4)	1.000	0.243	1.000	0.482
	≥0.071	80 (37.9)	77 (32.6)	1.260 (0.855-1.862)		1.160 (0.770-1.740)	
≥50	<0.071	174 (60.0)	207 (70.6)	1.000	0.007	1.000	0.007
	≥0.071	116 (40.0)	86 (29.4)	1.600 (1.140-2.270)		1.610 (1.140-2.280)	
Molecular subtype							
Luminal A	<0.071	105 (61.4)	366 (69.2)	1.000	0.060	1.000	0.080
	≥0.071	66 (38.6)	163 (30.8)	1.410 (0.983-2.018)		1.380 (0.959-1.988)	
Luminal B	<0.071	113 (61.1)	366 (69.2)	1.000	0.044	1.000	0.046
	≥0.071	72 (38.9)	163 (30.8)	1.430 (1.010-2.020)		1.440 (1.000-2.060)	
HER-2	<0.071	42 (64.6)	366 (69.2)	1.000	0.454	1.000	0.620
	≥0.071	23 (35.4)	163 (30.8)	1.230 (0.706-2.094)		1.150 (0.650-1.990)	
Basal-like	<0.071	29 (51.8)	366 (69.2)	1.000	0.009	1.000	0.014
	≥0.071	27 (48.2)	163 (30.8)	2.090 (1.190-3.650)		2.030 (1.150-3.560)	

OR, odds ratio; 95% CI, 95% confidence intervals.

[a] ORs were calculated based on the methylation levels of CpG sites increased per standard deviation, and were adjusted for age, BMI, race, location, family history of other cancer and family history of breast cancer.

Table S16. The association between methylation of the CpG located on genomic coordinate 2951137 in *PHLDA2* region and the risk of breast cancer in different subgroups.

				Univariate analysis		Multivariate analysis	
**Subgroup**	***PHLDA2*_2951137**	**Case (%)**	**Control (%)**	**OR (95% CI)**	***p***	**OR (95% CI)[1]**	***p***
Age (year)							
<50	<0.028	140 (66.4)	175 (74.2)	1.000	0.072	1.000	0.101
	≥0.028	71 (33.6)	61 (25.8)	1.450 (0.968-2.192)		1.430 (0.933-2.198)	
≥50	<0.028	183 (63.1)	214 (73.0)	1.000	0.010	1.000	0.009
	≥0.028	107 (36.9)	79 (27.0)	1.580 (1.120-2.260)		1.600 (1.130-2.290)	
Molecular subtype							
Luminal A	<0.028	111 (64.9)	389 (73.5)	1.000	0.031	1.000	0.033
	≥0.028	60 (35.1)	140 (26.5)	1.500 (1.040-2.170)		1.500 (1.030-2.180)	
Luminal B	<0.028	115 (62.2)	389 (73.5)	1.000	0.004	1.000	0.011
	≥0.028	70 (37.8)	140 (26.5)	1.690 (1.180-2.410)		1.610 (1.110-2.320)	
HER-2	<0.028	43 (66.2)	389 (73.5)	1.000	0.209	1.000	0.247
	≥0.028	22 (33.8)	140 (26.5)	1.420 (0.809-2.437)		1.400 (0.781-2.446)	
Basal-like	<0.028	36 (64.3)	389 (73.5)	1.000	0.142	1.000	0.118
	≥0.028	20 (35.7)	140 (26.5)	1.540 (0.851-2.730)		1.600 (0.875-2.878)	

OR, odds ratio; 95% CI, 95% confidence intervals.

[a] ORs were calculated based on the methylation levels of CpG sites increased per standard deviation, and were adjusted for age, BMI, race, location, family history of other cancer and family history of breast cancer.

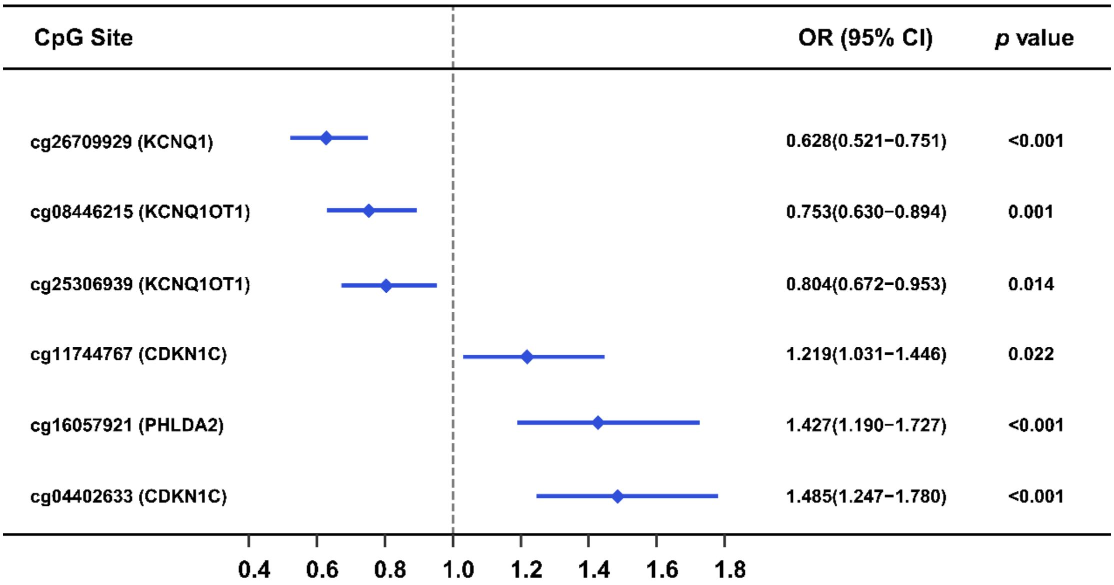

Figure S1. The association between 6 CpG sites of imprinted genes and the risk of breast cancer in GSE51032 dataset. OR, odds ratio; 95% CI, 95% confidence intervals. The forest plot showed the results of univariate logistic regression analyses of 6 CpG sites. ORs were calculated based on the increased methylation levels of CpG sites per standard deviation.

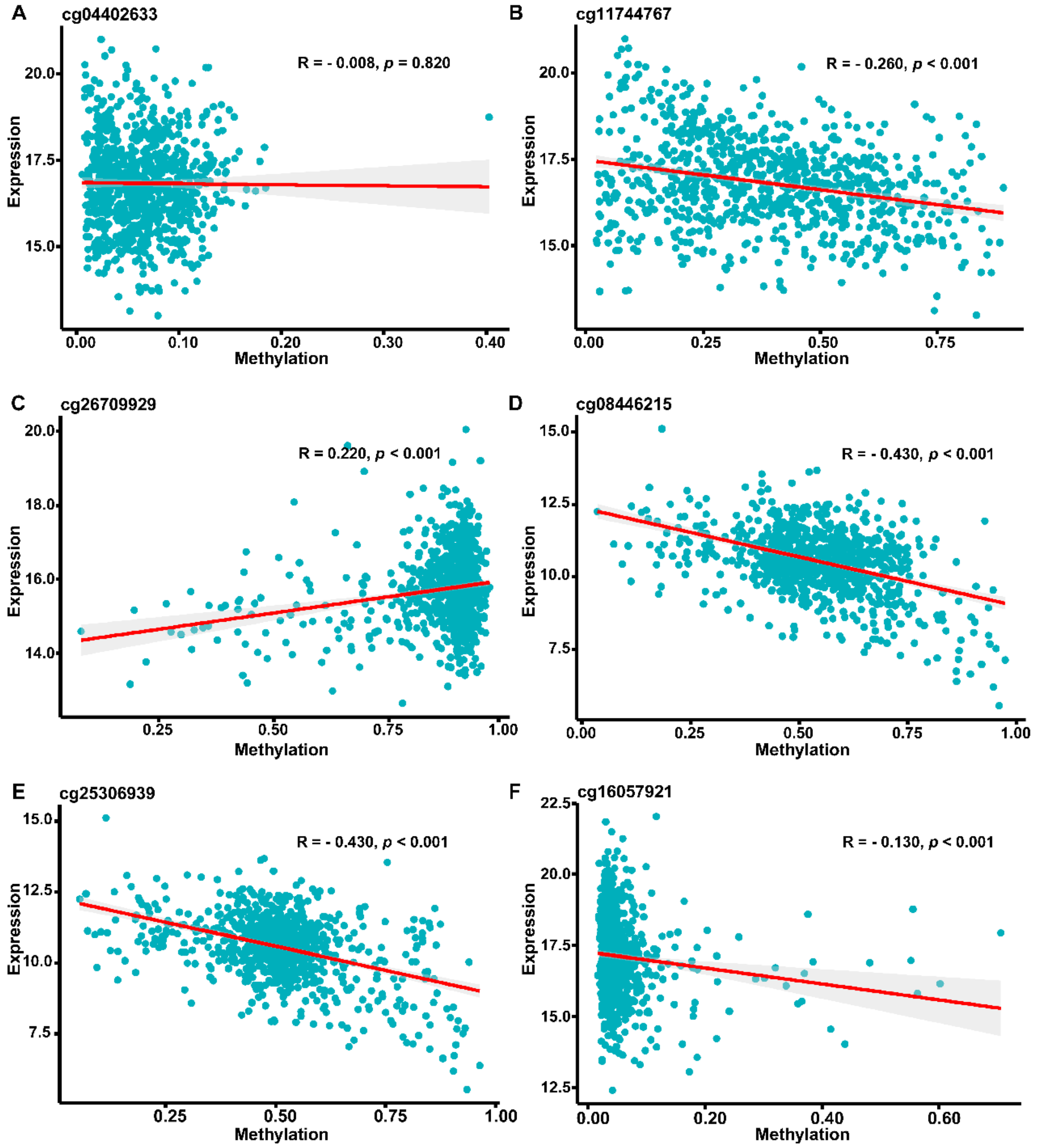

Figure S2. Correlation between methylation of 6 CpG sites and corresponding gene expression levels in TCGA dataset.

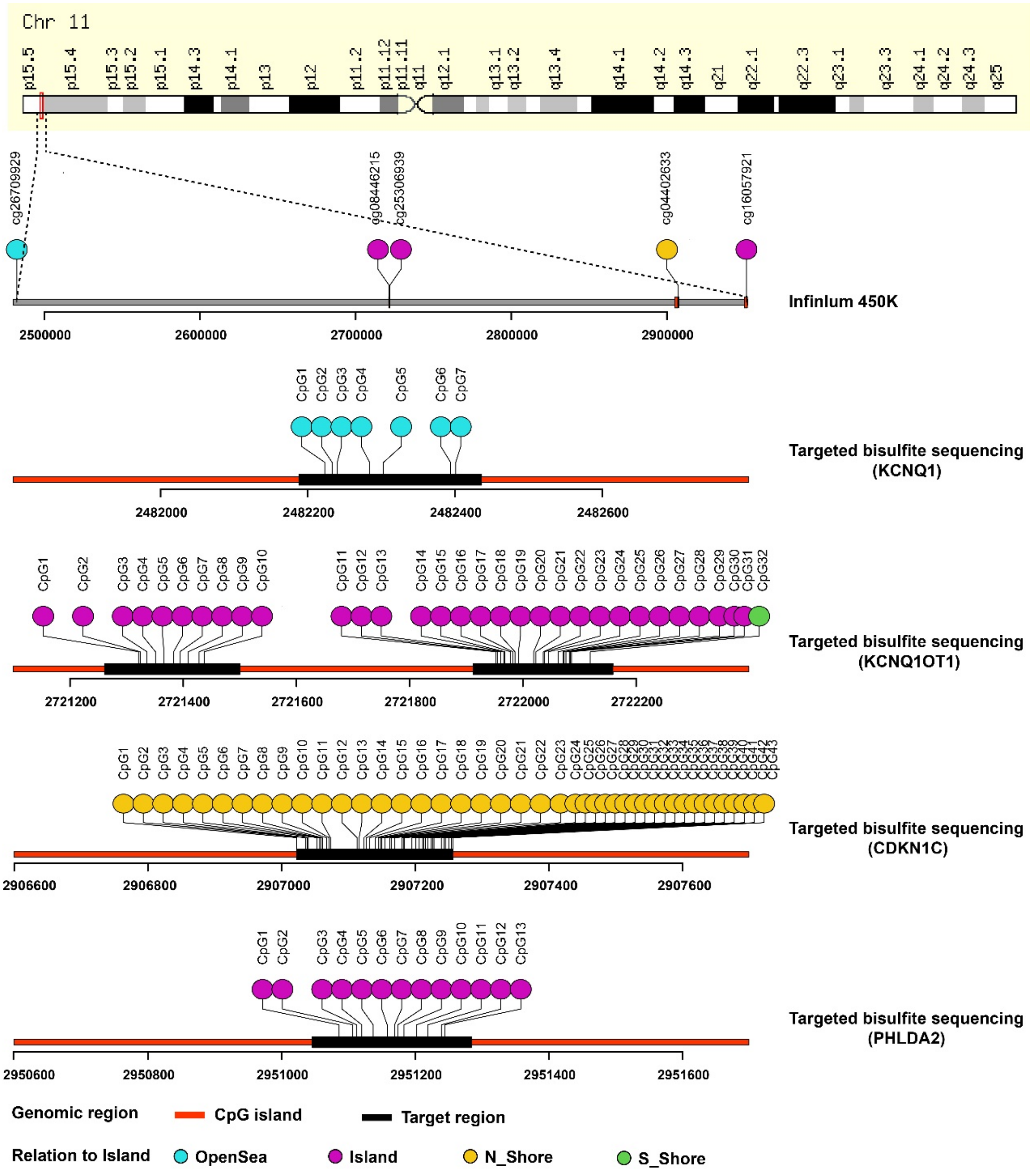

Figure S3. Target regions of imprinted genes for targeted bisulfite sequencing. In targeted bisulfite sequencing, five regions of *KCNQ1*, *KCNQ1OT1*, *CDKN1C*, and *PHLDA2* were amplified using primers designed based on the genomic location of cg26709929, cg08446215, cg25306939, cg04402633, and cg16057921. The target region of *KCNQ1* covered 7 CpG sites (CpG1 to CpG7), and CpG3 on *KCNQ1* region corresponded to cg26709929. The target region 1 of *KCNQ1OT1* covered 10 CpG sites (CpG1 to CpG10), and CpG5 on *KCNQ1OT1* region corresponded to cg08446215. The target region 2 of *KCNQ1OT1* covered 22 CpG sites (CpG11 to CpG32), and CpG31 on *KCNQ1OT1* region corresponded to cg25306939. The target region of *CDKN1C* covered 43 CpG sites (CpG1 to CpG43), and CpG12 on *CDKN1C* region corresponded to cg04402633. The target region of *PHLDA2* covered 13 CpG sites (CpG1 to CpG13), and CpG7 on *PHLDA2* region corresponded to cg16057921.

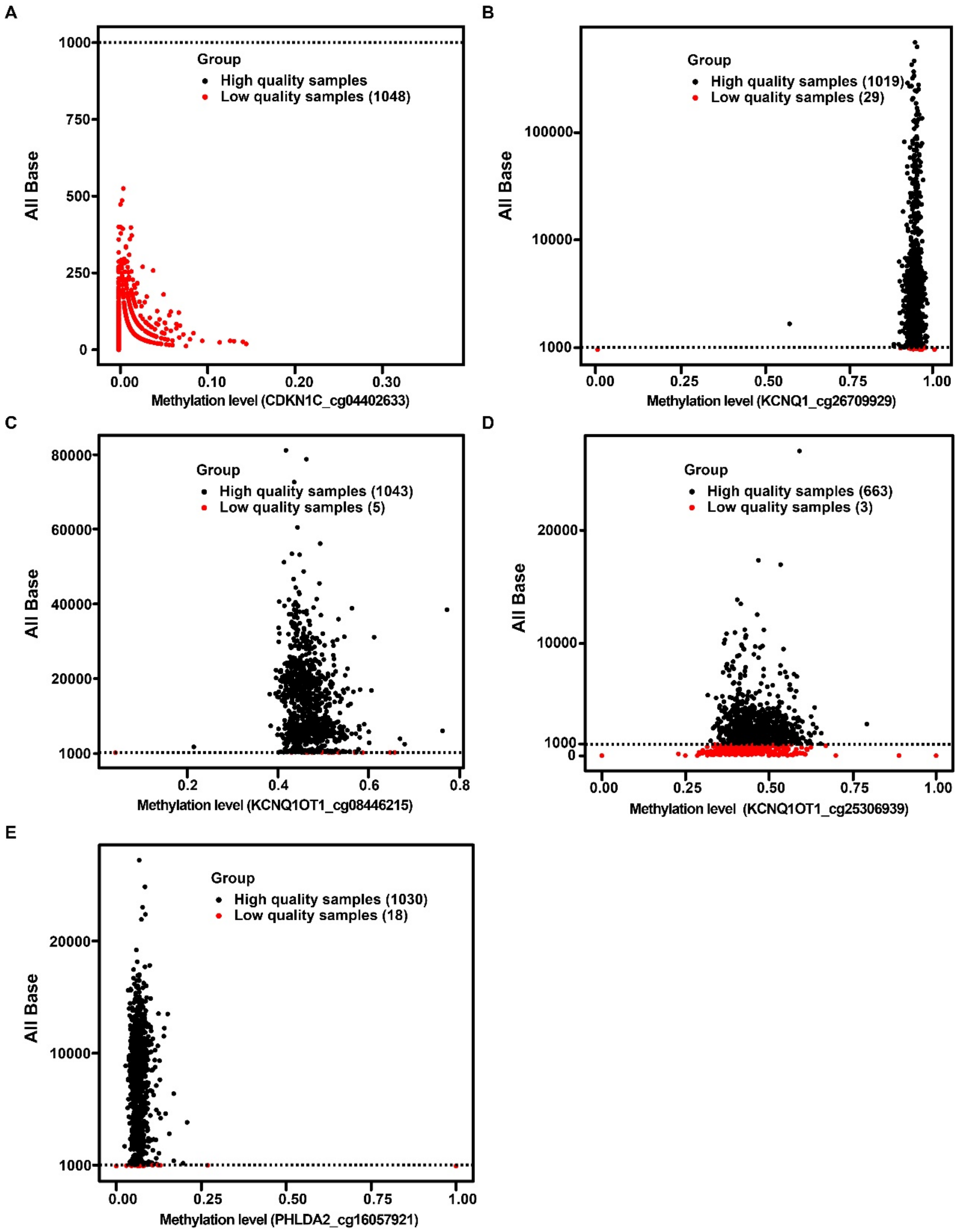

Figure S4. Quality control of samples based on different target regions. (A), (B), (C), (D), (E) represented the sequencing depth of cg04402633, cg26709929, cg08446215, cg25306939, and cg16057921 in targeted bisulfite sequencing, respectively.

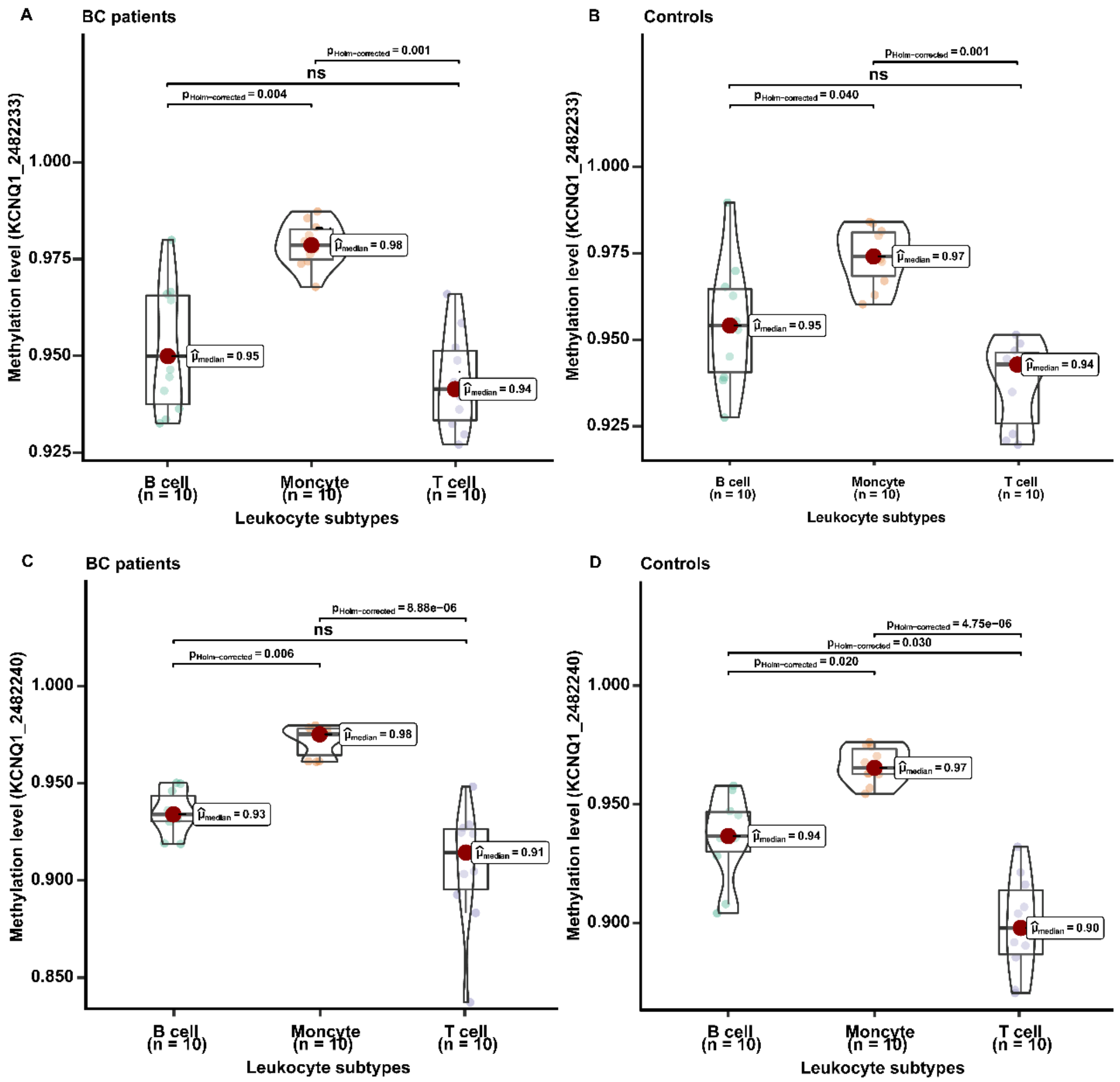

Figure S5. The methylation levels of the CpG located on genomic coordinate 2482233 and 2482240 in *KCNQ1* in T cells, monocytes, and B cells. The methylation level was measured in the samples (bisulfite-converted DNA from T cells, monocytes, and B cells) from 10 breast cancer patients and 10 healthy controls. The methylation levels between different leukocyte subtypes were compared by *Kruskal-Wallis* test.

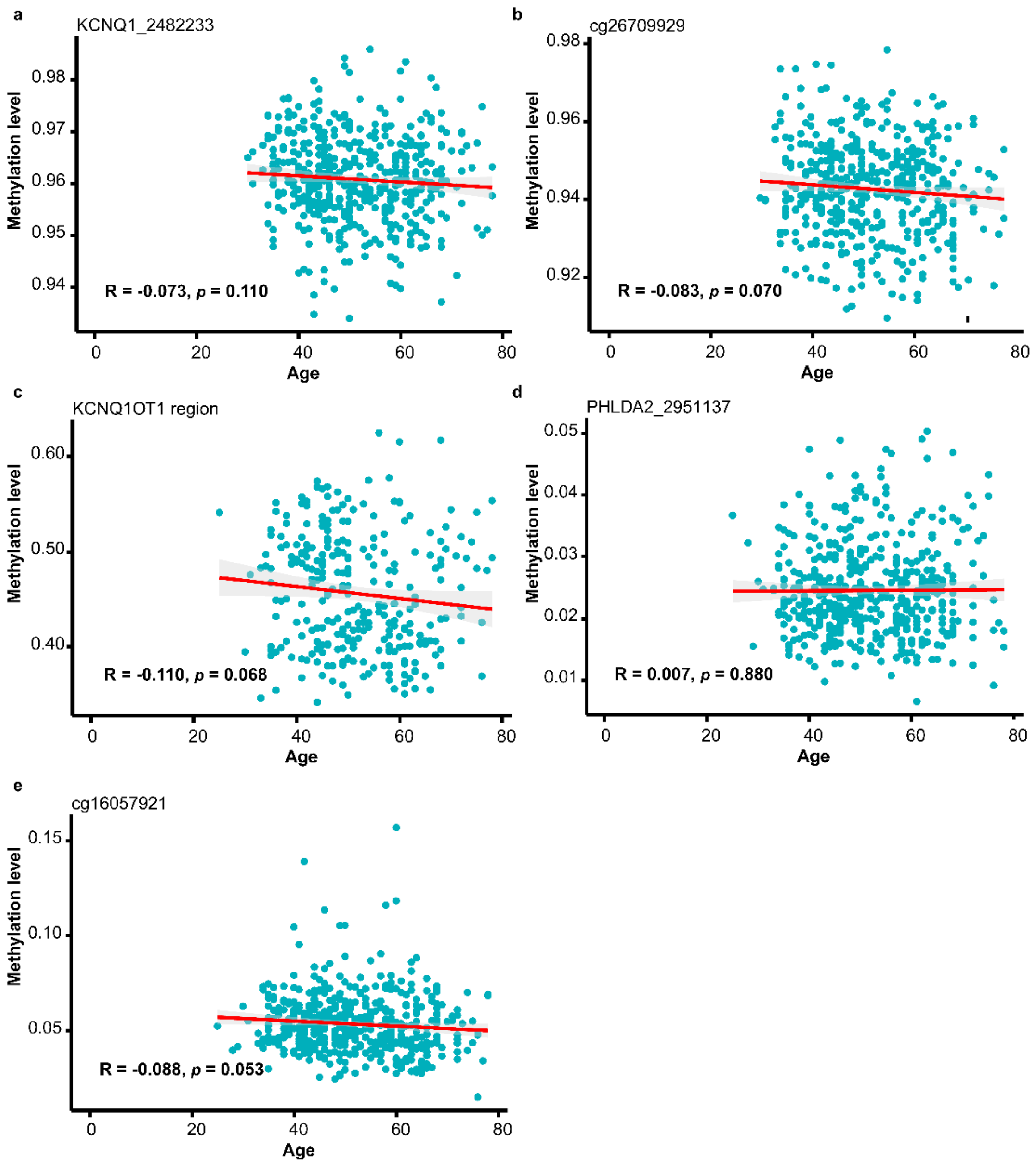

Figure S6. Correlation between methylation of *KCNQ1*, *KCNQ1OT1*, and *PHLDA2*, and the age of breast cancer-free individuals in the validation dataset.
